# ExprAlign - the identification of ESTs in non-model species by alignment of cDNA microarray expression profiles

**DOI:** 10.1186/1471-2164-10-560

**Published:** 2009-11-26

**Authors:** Weizhong Li, Andrew Y Gracey, Luciane Vieira Mello, Andrew Brass, Andrew R Cossins

**Affiliations:** 1 Centre for Genome Research, School of Biological Sciences, University of Liverpool, Crown Street, Liverpool, L69 7ZB, UK; 2 Marine Environmental Biology, University of Southern California, Los Angeles, CA 90089, USA; 3 Computing Science Department, University of Manchester, Kilburn Building, Oxford Road, Manchester, M13 9PL, UK; 4 EMBL - European Bioinformatics Institute, Wellcome Trust Genome Campus, Hinxton, Cambridge, CB10 1SD, UK

## Abstract

**Background:**

Sequence identification of ESTs from non-model species offers distinct challenges particularly when these species have duplicated genomes and when they are phylogenetically distant from sequenced model organisms. For the common carp, an environmental model of aquacultural interest, large numbers of ESTs remained unidentified using BLAST sequence alignment. We have used the expression profiles from large-scale microarray experiments to suggest gene identities.

**Results:**

Expression profiles from ~700 cDNA microarrays describing responses of 7 major tissues to multiple environmental stressors were used to define a co-expression landscape. This was based on the Pearsons correlation coefficient relating each gene with all other genes, from which a network description provided clusters of highly correlated genes as 'mountains'. We show that these contain genes with known identities and genes with unknown identities, and that the correlation constitutes evidence of identity in the latter. This procedure has suggested identities to 522 of 2701 unknown carp ESTs sequences. We also discriminate several common carp genes and gene isoforms that were not discriminated by BLAST sequence alignment alone. Precision in identification was substantially improved by use of data from multiple tissues and treatments.

**Conclusion:**

The detailed analysis of co-expression landscapes is a sensitive technique for suggesting an identity for the large number of BLAST unidentified cDNAs generated in EST projects. It is capable of detecting even subtle changes in expression profiles, and thereby of distinguishing genes with a common BLAST identity into different identities. It benefits from the use of multiple treatments or contrasts, and from the large-scale microarray data.

## Background

Transcript screening investigations have traditionally been led by sequence analysis of cDNA clone collections to define the identity of hybridisation probes included on microarrays for expression profiling [1]. Despite this, all eukaryotic EST collections contain large proportions of transcripts (~50%) that remain unidentified by unattended BLAST protocols. Some of these may represent new, undiscovered protein-coding or non-protein-coding transcripts [2-4]. Others may arise from untranslated regions of coding sequence RNA, which being non-conserved fail to align to reference databases. Finally, some may be concatenated constructs generated artefactually during the production of cDNA libraries.

We have experienced these kinds of difficulty in our analysis of ESTs from the common carp, *Cyprinus carpio *L., a well-used model species for research into environmental responses [5], and which is the subject of a substantial aquaculture interest for both food and ornamental uses. The common carp genome is widely thought to have become duplicated within the previous 12-15 Mya, and many duplicate paralogs are retained [6-8] to complicate the analysis. We originally generated a medium-scale collection of ~13.5K directional, cDNA clones from multiple tissues [9], though this has more recently been increased [10]. 9,202 directional EST were assembled into 6,033 transcriptional units. Of these only 3,252 were BLAST-identified leaving 2,701 as unclassified, many of which displayed interesting expression properties in response to a range of chronic stress treatments [9].

Additional information regarding the identity of ESTs may come from the comparison of expression profiles of one microarray probe with another since different probes arising from the same gene should have very highly correlated profiles whilst probes with an identical BLAST identity but arising from different members of a gene family might present divergent expression profiles. Either way, co-expression indices can be used as evidence in seeking an identity for a BLAST-unidentified cDNA clone, and can separate putative isoforms. To explore the limits of expression profiling, and the extent to which dissimilar but co-regulated genes may confound the process, we have accumulated data from a very large number of microarray hybridisations, including RNA from all of the major organs of common carp exposed to a range of environmental stresses, including chronic cooling [9], chronic hypoxia [11] and starvation/refeeding protocols. This large dataset represents a substantial data resource that can be used to suggest gene identity through correlation analysis. Here we describe the **Expr**ession **Align**ment (*ExprAlign*) technique for assigning a putative gene identity, which, following the pioneering work of Kim *et al*. [12,13], is based on the clustering of gene expression profiles [14-17]. This resolves a number of issues relating to the identification of probes that were unidentified by conventional unattended BLASTx procedures, including those from untranslated regions of transcripts.

## Methods

### ESTs resource and common carp microarray data

We used the EST resources from carpBASE 2.1, which was constructed by the EST analysis package EST-ferret 2.1 http://legr.liv.ac.uk. This comprised the 13,349 directional cDNA clones already described [10], of which 9202 were 5' end sequenced, BLASTx identified and annotated with gene ontology, KEGG and CDD terms.

The cDNA microarray used in this work has been described in [9] and [11], and comprised 13440 PCR-amplified cDNA probes, including blanks and standards. The raw expression data has been deposited in ArrayExpress E-MAXD-1 and E-MAXD-10, respectively. The gene expression data used in this analysis comprised 707 common carp RNA samples, hybridised to 1414 cDNA microarrays, all using a reference-based, dye-swap design against a common reference using dye-swap, and with 4-fold or greater biological replication. These experiments were conducted with ethical approval and corresponding personal and project licences of the Home Office, U.K 189 RNA samples were generated from the study of chronic cold stress [9], including samples for time-course after transfer from a preconditioning temperature of 30°C to 23°C, 17°C, and 10°C. Tissues examined were brain, gill, heart, intestine, kidney, liver and skeletal muscle. 414 RNA samples were used in a time-course study of hypoxia stress [11] in 4 tissues (brain, heart, intestine, liver and skeletal muscle), conducted at two temperatures (17°C and 30°C). 104 samples were from a study of starvation over a 6-week time course during which the animals were starved and then re-fed (I. Hardevig and A.R. Cossins, unpublished). The 'starvation' data included samples for liver and skeletal muscle only. Normalisation and statistical processing of the resulting data has been previously described [9,11] using established techniques [18].

### Computing and filtering Pearson's correlation coefficients

The Pearson product-moment correlation coefficient (*r*) was calculated for every pair of array probes. To speed this up a programme called "CORR" http://legr.liv.ac.uk was written in C, resulting in completion in approximately 40 minutes using a single Linux machine. The Receiver Operating Characteristic (ROC) curves were implemented as described in Additional File 1, Section A, to optimize the threshold of the correlations [19]. The resulting criterion was used to filter the *r*-values, and the selected relationships were stored in a spreadsheet consisting of a few thousand gene-pairs (the rows), with coefficient scores.

The optimized threshold for filtering Pearson correlation coefficients was determined by plotting the sensitivity (True Positive, P^+^) of the comparison against the selectivity (False Positive, P^-^). The sensitivity P^+ ^indicates the probability of the observed true positives at a threshold and the selectivity P^- ^shows the probability for the observed true negatives at a threshold. On the other hand, the probability of the missed true positives was given by (1 - P^+^) and the probability of the missed true negatives by (1 - P^-^). The total probability of missing the true positives and true negatives was given by E = (1 - P^+^) + (1 - P^-^), this providing the optimal threshold. The calculations for P and E are detailed in the Additional File 1, Section A.

### Visualising expression alignments

The VxInsight package [20-22] was implemented for clustering gene expressions using the Pearson's correlation coefficients and to visualise the alignments. The VxInsight package consists of three parts: VxOrd, VxInsight and VxImport. VxOrd implements the force-directed ordination algorithm to assign the X, Y coordinates for each gene on a 2-dimensional surface based on the clone pair similarities of correlation coefficients. Then the coordinate maps were converted to the 3-dimensional mountain terrains in VxInsight. Finally, VxImport loaded the gene annotation from carpBASE 2.1 into VxInsight for biological interpretations.

**Figure 1:**
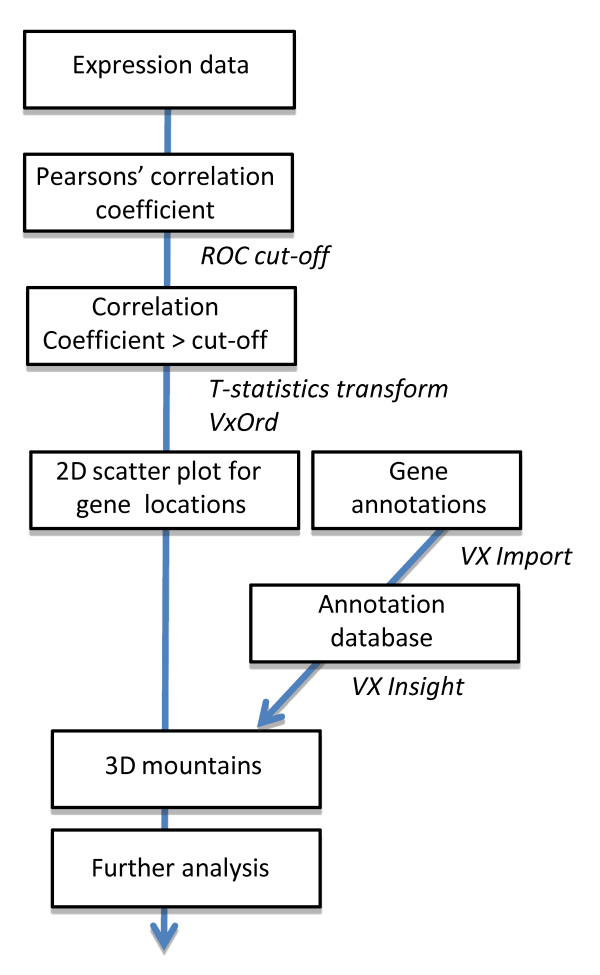
**Flow diagram depicting the order of events (in boxes) and informatic tools and packages used (italics) in the *ExprAlign *pipeline**.

Figure 1 illustrates the stages used in *ExprAlign *to generate a landscape depiction of the expression profile, starting from the microarray intensity data and ending with the production of the colour-rendered 3D landscape.

### Post ExprAlign analysis

Groups of cDNA clones included within landscape features were assessed using BLASTx, searches as described previously [10] followed by mapping of FASTA sequences onto the zebrafish genome v8 as described by Christoffels *et al*. [23], or onto common carp fosmid clones BX571686 and BX571725 [24]. Details of Pearsons correlation coefficient of closely related clones, and of mountain allocations, have been included as part of the clone entry in carpBASE2.1.

## Results

### Correlation analysis

Using normalised data generated from 707 RNA preparations from the cold and hypoxia datasets, each containing ~13,440 cDNA probes from multiple tissues we calculated the Pearson correlation coefficient [25] between every pair of probes represented on the microarray. This required ~180 million calculations, covering different combination of stress and time, as well as all different tissues. We then used a Receiver Operating Characteristic (ROC) procedure [19] to optimize the threshold that minimises the representation of false positives at 0.858. The coefficients below this value were discarded leaving 30868 correlation coefficients representing 3039 gene probes for construction of the landscape.

### Network analysis using 3D landscape for visualisation

**Figure 2:**
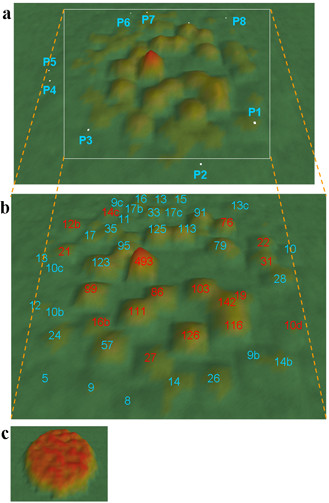
**A landscape representation of the co-expression profile for the GE analysis of common carp genes**. (a) An overview landscape. The white spots, P1 to P8, represent the locations of different parvalbumin isoforms. (b) shows a magnified part of (a) showing labels for each feature displaying the number of clones within that feature. 'Identified' mountains were indicated in blue text and 'unknown' mountains in red. (c) Terrain map derived from permuted and thus random-shuffled array data.

We then created a network representation of the *r*-values that exceeded the ROC threshold, which was ordinated and visualized using VxInsight [20]. This clustered groups of gene probes with high *r*-values between them using a force-repulsion model and represented them as features in a 3-dimensional landscape metaphor, as an aid to easy interpretation (see Figures 2a and 2b). The height of each landscape feature was an indication of the number of gene probes contained within it, and large-scale features ('mountains', 'hills') can include a number of smaller features ('hillocks'). The 3039 gene probes previously identified comprised 1192 possessing a BLASTx identity whilst the remaining 1847 were unclassifiable. Big mountains were located around the centre of the landscape while smaller mountains were positioned away from the centre. The distance between each feature, both large and small, was representative of the *r*-values that connect the features. Thus a small distance between landscape features indicates that each cluster of gene probes was more similar to each other than if they were more distantly linked.

To assess the significance of the topographical patterns revealed by this process, we permuted the expression table by shuffling the values for all probes across all arrays. The resulting *r*-values were uniformly low with only 3 pairs of probes with *r *> 0.858, and 33870 with *r *> 0.25. We used the latter data to generate a landscape shown in Figure 2c, which showed no structuring. This indicates that the landscape features evident in Figure 2a do not result from random effects in the underlying data, and represent biologically significant outcomes.

### Dependency of landscape features upon scale and diversity of datasets

To determine whether the structure of the gene co-expression landscape and the clustering of genes was influenced by the particular dataset used in their construction, we recalculated the landscape using only the data from the 386 arrays generated for the cooling experiment [9]. In this case 4236 microarray probes exceeded the ROC threshold and were ordinated onto the landscape map, comprising 1776 identified and 2460 unclassifiable gene probes. 46 mountains (labeled 'CE', 'cold expression') were generated of which 22 were identified.

**Figure 3:**
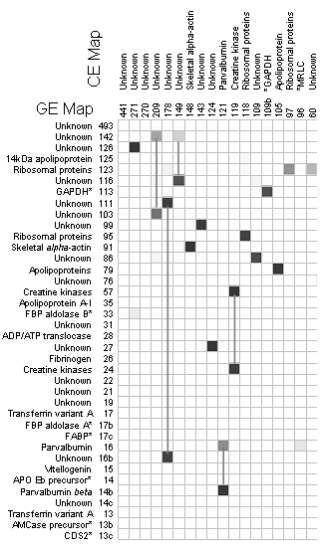
**Matrix to compare the distribution of selected gene identities in GE and CE landscape co-expression features**. Additional File 1, Figure S1 comprises all features. The labels for each column or row indicates the number of genes within that landscape feature. The GE features are listed in Table 1. The numbers contained within individual cells indicate the number of microarray probes common to the linked GE and CE features. The denser colour represents a greater level of the agreement.

We then compared the landscape features for both the GE and CE datasets (provided in reduced form in Figure 3, and shown in full in Additional File 1, Figure S1) using a matrix in which cells contained the number of gene probes that were included in corresponding landscape features. We found 21 highly similar mountain-pairs between GE and CE mountains, indicated by greyed cells. However, we noted some differences between the two landscapes. Thus, the clones in mountain CE209 were separately located in GE142 and GE103; clones in CE178 were separately located in GE111 and GE16b; and the clones in CE149 were separately located in GE142 and GE116. Feature CE26 (see Additional File 1, Figure S1) was linked to three GE features, namely GE35 (apolipoprotein A-I), GE17 (transferrin) and GE13 (transferrin). Moreover, CE119 (creatine kinases) was linked to GE57 and GE24 (both labelled as creatine kinases); and CE121 (parvalbumins) were broken down to GE16 and GE14b (both parvalbumins). The splitting of CE features into two GE features might indicate the separation of isoforms.

**Figure 4:**
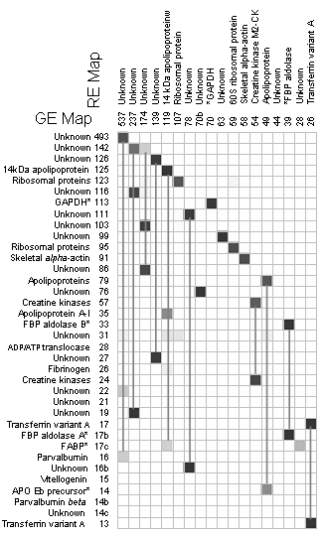
**Matrix for agreement and difference of global mountains and random mountains**. This figure includes selected landscape features whilst Additional File 1, Figure S2 comprises all features. Other details are as described in the legend to Figure 3.

We conclude that the discrimination between closely linked gene clusters was significantly affected either by the scale or by the diversity of the data used in their construction. We then tested which of these was the important factor by randomly discarding 50% of the arrays included in the GE dataset. The resulting landscape contained 2444 clones in 27 mountains (labelled 'RE', randomised expression) of which 12 were identified. We then compared the contents of landscape features of the RE dataset with the original GE dataset (summarised in Figure 4 and displayed in full in Additional File 1, Figure S2). This shows that 17 of the RE mountains were related to single features on the GE landscape, and that the 'identity' of these features was conserved. However, 11 of the RE mountains were linked to multiple GE mountains; for example, RE237 and RE174 were each linked to 3 different features in the GE landscape. Thus, whilst the main features of the landscape were robust to reductions in the amount of array data some were sensitive to increasing data complexity, as produced by undertaking more diverse experimental treatments.

### Relating unidentified or unclassifiable clones to identified genes

To test the significance of the representation of identified genes within each of the landscape features we computed *p*-values for the GE dataset for each gene identity using Fisher's exact test. Table 1 lists the *p*-values, all of which lay between 10^13 ^and 10^101^, indicating highly significant enrichment of the identified gene within the mountain.

**Table 1:** Summary of landscape features identified for the complete (GE) dataset.

GE Mountain i.d.	No. of identified clones	Best represented gene identities						No. of relatable unknown clones
			
		Protein description	No. in mountain	% of the identified clones in mountain	% of clones in mountain	No. in carpBASE 2.1	*p *to the carpBASE 2.1	
**125**	48	14 kDa apolipoprotein	34	70.8	27.2	65	3.2E-60	77
**123**	53	Ribosomal proteins	39	73.6	31.7	339	5.9E-38	70
**113**	55	Glyceraldehyde-3-phosphate dehydrogenase	50	90.9	44.2	70	6.3E-101	58
**95**	23	Ribosomal proteins	19	82.6	20	339	1.2E-20	72
**91**	46	Skeletal alpha-actin	36	78.3	39.6	71	6.5E-65	45
**79**	39	Apolipoproteins	33	84.6	41.8	113	3.9E-53	40
**57**	24	Creatine kinases	20	83.3	35.1	74	5.9E-36	33
**35**	15	Apolipoprotein A-I	13	86.7	37.1	47	8.9E-27	20
**33**	28	Fructose-bisphosphate aldolase B	26	92.9	78.8	30	4.1E-65	5
**28**	26	ADP/ATP translocases	26	100	92.9	43	1.7E-60	2
**26**	16	Fibrinogen	14	87.5	53.8	35	4.0E-31	10
**24**	6	Creatine kinases	4	66.7	16.7	74	3.E-07	18
**17**	9	Transferrin variant A	8	88.9	47.1	44	4.1E-17	8
**17b**	15	Fructose-bisphosphate aldolase A	13	86.7	76.5	30	7.6E-30	2
**17c**	13	Fatty acid-binding protein	13	100	76.5	28	2.3E-32	4
**16**	9	Parvalbumins	8	88.9	50	114	1.4E-13	7
**15**	6	Vitellogenin	6	100	40	10	3.6E-18	9
**14**	5	Apolipoprotein Eb precursor	5	100	35.7	19	2.0E-13	9
**14b**	14	Parvalbumins	14	100	100	114	4.5E-25	0
**13**	9	Transferrin variant A	7	77.8	53.8	44	2.8E-14	4
**13b**	7	Acidic mammalian chitinase precursor	7	100	53.8	11	6.8E-21	6
**13c**	10	Carp Desaturase 2 (CDS2)	10	100	76.9	15	2.2E-28	3
**12**	9	Troponin T, fast skeletal muscle isoforms	9	100	75	22	2.1E-23	3
**11**	9	Apolipoprotein C-I precursor	9	100	81.8	24	5.6E-23	2
**10**	5	Myoglobin	5	100	50	6	1.0E-16	5
**10b**	8	Warm-temperature-acclimation-related-65 kDa-protein	5	62.5	50	15	2.8E-12	2
**10c**	9	Uncoupling protein 1	9	100	90	10	4.3E-28	1
**9**	8	C-type lectin	8	100	88.9	19	2.1E-21	1
**9b**	9	Invariant chain like protein 2	9	100	100	20	7.1E-24	0
**9c**	6	Elongation factor 1-alpha; EF-1-alpha	6	100	66.7	13	3.0E-17	3
**8**	6	Alcohol dehydrogenase	6	100	75	24	2.3E-15	2
**5**	4	RING finger protein 28	4	100	80	6	3.0E-13	1

**Total**	**549**							**522**

Column 1 represents the number of gene probes included within the landscape feature indicated in Figure 2, and this is also used to denote the identity. Column 2 indicates how many of these were BLAST identified as indicated in column 3. The number of the best-represented identified gene in a mountain must be >2, and its percentage of clones in mountain must be over 20%. *P *represents the significance (Fishers Exact test) of the gene being over-represented in the mountain comparing to the whole clone set included within carpBASE 2.1.

Secondly, we examined individual clusters either by attended curation of the ESTs followed by BLASTx or by mapping clones onto the zebrafish genome. Thus, for mountain GE13c, only 4 clones recovered a BLASTx hit for the common carp desaturase-2, 2 of them following a frame shift correction. A further 6 were confirmed as belonging to this gene using BLASTn against the 2 available common carp fosmid clones but in the untranslated region, two of them being very short sequence reads. The three remaining clones failed to provide a sequence, but were inferred as being the same gene. Mountain GE10d contained only unidentifiable sequences (thus, not included in the listing of identified mountains in Table 1), all of which belonged to the same carpBASE2.1 contig (1127-2) and which mapped to the same zebrafish location (Chromosome 23: 24767100). This lies within an unprocessed pseudogene (locus i.d. OTTDARG00000030773) so whilst the identity and status of this transcriptional product remains unclear, it is revealed as being expressed in particular common carp tissues and in response to particular environmental influences. Mountain GE10 contained 5 ESTs that were identified by BLASTx as myoglobin, and 5 ESTs that were frame shift corrected also to BLASTx as myoglobin. Finally, in mountain GE113, 50 of the 55 identified genes (i.e. ~91%) were BLASTed as glyceraldehyde 3-phosphate dehydrogenase. The *p*-value for this identity (6e-101) was very highly significant. However, 5 ESTs showed homology to different genes including microglobulin, adenylate kinse, 60S ribosomal protein, and a glycoprotein precursor. The remaining 58 clones lacking sequence data were thus inferred as having the same identity as the predominant gene.

For large mountains, in particular, we found multiple identified genes being represented, making it difficult to ascribe a single identity to unidentified clones. In some cases we have generated 3-5 sub-clusters using the K-means technique, each of which displayed a more singular gene identity with which to infer an identity for the unidentified or un-sequenced ESTs (see below).

Using these methods, we inferred a tentative identity for a total of 522 unidentified ESTs, which represented 17% of the 3039 unidentified ESTs. These are listed in Additional File 1, Table S1. From this it is evident that many clones failed to generate sequence data during the automated sequence analysis, yet they provided informative amplicons for array fabrication.

### Discrimination of gene isoforms by ExprAlign

**Figure 5:**
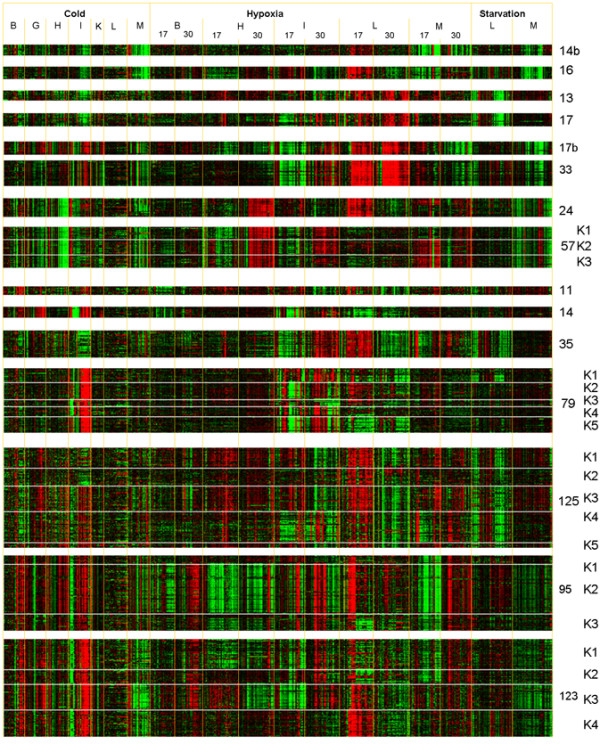
**Heatmaps for identified GE mountains incorporating data for 707 RNA preparations comprising stress treatments in up to 7 different tissues**. The numbers on the right side indicates the mountain names which corresponds to the number of gene probes. K1, K2, ⋯, Kn shows the sub-clusters generated by the *K*-means clustering technique for each mountain. B - brain, G - gill, H - heart, I intestine, K kidney, L liver, and M - muscle. Red and green indicates up- and down-regulated gene expressions respectively. Heatmaps for additional identified GE mountains are shown in Additional File 1, Figure S3.

We have explored the extent to which *ExprAlign *supplements sequence alignment in distinguishing isoforms with gene families. Figure 5 (and Additional File 1, Section B) shows for a number of co-expression mountains the changes in transcript expression of constituent clones as a conventional heatmap across all three experimental conditions.

#### (i) Fructose-bisphosphate aldolases

Vertebrates possess three tissue-specific isoforms of fructose-bisphosphate aldolase [26-28]: A (muscle and red blood cells), B (liver, kidney, stomach and intestine) and C (brain, heart and ovary) [29]. Assembly of our common carp ESTs identified these three 3 isoforms: S341 (the prefix 'S' signifies an assembled sequence group from carpBASE 2.1; aldolase A), S488 (aldolase B) and S698 (aldolase C).

Aldolase A and B were separately resolved into two neighboring mountains: GE17b containing only S341 (isoform A) and GE33 containing only S488 (isoform B). The close proximity of these mountains in Figure 2 was indicative of the similarity of their expression patterns (see Figure 5), yet *ExprAlign *cleanly resolved even these subtly different expression patterns in precise agreement with the sequence alignments. Figure 5 shows that resolution between isoforms depended mainly on expression differences in the chronic cold-exposure experiment, and in just 3 tissues; brain, gill, heart and kidney. Thus, aldolase A was up-regulated not only in hypoxia muscle (17°C) but also in hypoxia liver, hypoxia brain, hypoxia intestine (30°C), cold brain, cold gill, cold heart, cold intestine and cold kidney. Aldolase B was up-regulated in cold-intestine, cold heart, hypoxia muscle 17°C, hypoxia intestine 30°C and hypoxia liver. Previous work [29] compared the absolute level of the gene expression of aldolase isoforms, but our study focused on stress-regulated changes.

#### (ii) Parvalbumins

carpBASE2.1 contained 89 clones identified as parvalbumin. All of these were located in 8 entirely separate clusters that were positioned around the edge of the landscape (Figure 2a: P1 to P8), indicating very divergent expression patterns. Two distinct phylogenetic lineages for parvalbumins have previously been delineated: α and β [30,31], and manual sequence alignment a single α isoform (α1) and 8 β isoforms, (β1- β8), all of which are expressed as proteins [32]. The β6 isoform was located in P7 (GE16, see Figure 2b), P6 included β7, P3 included β5, whilst P1 (GE14b) contained β6, β7 and β1.

#### (iii) Transferrin variant A

Crucian carp (*C. auratus*) has two transferrin variants [33], whilst silver crucian carp (*C. auratus gibelio*) has 5 and white crucian carp (*C. auratus cuvieri*) has 3 [34]. We found that ESTs blasting to transferrin variant A were located in two mountains, GE13 and GE17 that were located in two separate locations but in the same sector of the landscape. Whilst overall the expression profiles (Figure 5) were similar differences were noted in intestinal and cardiac tissues exposed to hypoxia. However, sequence alignment of the ESTs in these two mountains failed to demonstrate any sequence clustering which related to the separate expression mountains. The lengths of the GenBank accessions for carp transferrin variants (AF457152, EU71532-EU71535) were over 2200 bp whilst our ESTs were ~400 bp of which only 200 bp could be used for sequence alignment. So it is most likely that the mountains contain different isoforms and that the sequence domains linked to these expression differences lay outside of the sequenced stretches.

#### (iv) Apolipoproteins

The apolipoprotein gene family were represented in five different mountains, namely GE11 (containing apolipoprotein C-I precursor, apoC-I), GE14 (apolipoprotein Eb precursor, apo-Eb), GE35 (apolipoprotein A-I, apoA-1), GE125 (14-kDa apolipoprotein, apo-14 kDa) and GE79 (mixed apolipoprotein & its precursor). Pufferfish apoA-I was expressed mainly in liver but apo-14 kDa was mainly expressed in liver and less abundantly in brain [35]. We show in common carp that some members of this gene family displayed hypoxia responses notably in the intestine (GE35, Figure 4). By contrast, the features GE11 and GE14 showed stress responses of apoC-I and apo-Eb genes in brain and intestine, respectively. The transcripts of the latter gene were also observed in gill.

~85% of BLAST-identified clones in GE79 were apolipoproteins (Table 1). Using the K-means method we sub-clustered the gene probes within GE79 into 5 clusters (K1 to K5, see Figure 5) which were linked to specific gene identities; thus, GE79-K3 was related to fatty acid-binding protein, GE79-K1 to apoA-I, GE79-K4 to apoEb precursor, and GE79-K2 and GE79-K5 were related to apoA-IV precursors. Mountain GE125 was split into 4 *K*-means sub-clusters: GE125-K3 and GE125-K4 were 14 kDa (fish-specified) apolipoproteins, GE125-K2 was related to other apolipoproteins, and GE125-K1 was unknown.

#### (v) Creatine kinases

Creatine kinases were located in mountains GE57 and GE24. Vertebrates typically express four isoforms: cytosolic muscle type (M-CK), cytosolic brain type (B-CK), mitochondrial ubiquitous, acidic type (Miu-CK), and mitochondrial sarcomeric, basic type (Mis-CK) and three M-CK sub-isoforms (M1-CK, M2-CK, and M3-CK) have been reported for common carp [36], and were confirmed proteomically [37]. *K*-means clustering split GE57 into 3 sub-clusters; GE57-K2 was designated M2-CK, and GE57-K3 as M3-CK. GE57-K1 contained unidentified ESTs, which might be M1 or other isoform.

## Discussion

Approximately 46% of the singletons and assembled contigs in our common carp EST project failed to yield an identity using the unattended BLASTx procedure [10], some of which represent non-overlapping, or 3' sequences of identified genes. Other clones failed to yield a sequence on automated analysis yet provided suitable hybridisation probes on the microarray. Given these special problems and complications in this species due to a recently duplicated genome, the sometimes incoherent nature of subsequent gene losses [24], and the divergent tissue- and response-specific expression patterns so generated [9], we have explored how expression alignment techniques might complement the more usual sequence alignment methods to assign an identity for an otherwise unidentifiable sequence.

Our approach was based on the idea that expression profiles for non-overlapping probes derived from a same gene should be highly correlated when tested across a range of experimental treatments, and this should enable unidentified clones to be identified by comparison with identified clones. Similarly, comparing expression profiles for cDNA microarray probes possessing the same BLASTx identity, offers a means of testing their common identity, given that they may represent unrecognised isoforms or variants of a given gene. Thus, combining alignment of sequence data with that from gene expression data offers a useful means of improving the quality of gene identification, and for discriminating isoforms or members of gene families whose separate identify may not easily be made evident using conventional methods.

For this work we chose to include all available cDNA clones on the microarray, resulting in up to 80 clones per contig, and to gather expression profiles from a wide range of major organs and tissues, exposed to a range of experimental treatments. Consequently, the carp array included the substantial repetition of some genes and this provided greater support for the identified gene clusters. Our approach was based on the comparison of the expression profiles of pairs of probes using Pearsons correlation coefficients which were used to create a network linking genes together on the basis of their shared expression properties. The VxInsight algorithm uses a force-repulsion mechanism to gather the distributed gene networks into discrete clusters, which are then presented in an easy-to-understand landscape metaphor.

We show that the resulting landscape features, and the associated clusters were robust, first, because permuting and randomising the expression values generated neither high correlation coefficients nor landscape features, and second, because the form of the clusters are largely retained when using different scales of array data from small to large. We show that datasets that contain a wider range of experimental treatments and tissues can fragment the gene clusters into smaller forms, each with a distinctive character. Thus, the exact level of discrimination achieved depends upon the diversity of the data used in its construction, with extra experimental treatments offering additional changes in the expression relationships between genes, thereby refining the resulting correlations.

### Gene identification using ExprAlign

Many of the resulting landscape features or mountains generated by VxInsight contained predominantly just one kind of BLAST-identified gene, and we show that there is substantial enrichment of these genes within the features compared to chance alone. Thus unidentified probes within that mountain were also tentatively labelled with that gene identity. Using this approach we were able to impute an identity to 522 unidentified clones in the GE landscape, which represented ~17% of all unidentified clones on the map. The validity of this assignment can be tested by the attended analysis of the clone sequences. This was achieved for mountain GE10 which possessed 5 different probes identified as myoglobin by BLASTx, and another 5 probes lacking an identify. Closer inspection of the corresponding sequences, and manual attempts at alignment, were subsequently able to demonstrate that all of the unidentified ESTs were also myoglobin, including additional examples of a unique brain-specific isoform [11]. This indicates the limitations of unattended BLAST analysis, and where appropriate, the need for manual verification of clones assigned with an *ExprAlign *identity. A similar result was obtained for parvalbumin [32].

### Separation of isoforms using ExprAlign

*ExprAlign *has also proved useful in separating clones that have been assigned a common BLASTx identity but which have distinctive expression profiles. If clones possessing the same gene BLAST identity were indeed sourced from the same transcriptional start site, then they should display identical expression patterns. On the other hand, if the clones were representative of different isoforms with distinctive expression properties, then they would occupy different features on the expression landscape, or could be distinguished using additional clustering techniques. We show that these expectations are largely met for a series of test cases, as described above. As a further example, in the case of fatty acid-binding protein (mountain GE17c, not shown), we identified 13 cDNA clones all possessing the same BLASTx sequence alignment, but which displayed two contrasting expression profiles. In this particular case, the differences were very subtle and limited to just one tissue (liver) responding to just one treatment condition (hypoxia at 17°C). Separation required application of K-means clustering to the genes contained within the landscape feature. This again indicates that the level of functional dissection possible by *ExprAlign *depends to a significant extent on the diversity of treatments for which array data is generated.

## Conclusion

By leveraging large-scale microarray data, the *ExprAlign *approach offers a practical aid to gene identification, and for the discovery of novel isoforms or variants of known genes. It bears comparison with the more conventional sequence alignment techniques in that both depend upon an association of co-expression properties, or sequence alignment, respectively, with a set of known standards. Both are limited by the available data, and both have limitations. We show that *ExprAlign *identifies subtle differences in expression properties which may not be evident from sequence similarity, particularly if the sequence data is limited to the 800 bp provided by a typical single pass read or if clones fail to generate sequence in automated procedures. Finally, it reveals ESTs possessing the same BLAST identity but which have distinctive expression profiles.

## Authors' contributions

WL undertook the collation and analysis of microarray data and contributed to drafting the manuscript. LVM undertook sequence alignment and mapping onto zebrafish, ARC and AYG both conceived of the work, obtained funding, supervised the implementation and drafted the manuscript. AB participated in the design of the study particularly to the form of the data analysis, the development of the ROC procedure, and the infrastructure for large-scale computations.

## Supplementary Material

Additional file 1 **ExprAlign - the identification of ESTs in non-model species by alignment of cDNA microarray expression profiles**. This document contains supplementary material for the abovementioned paper. It includes the following Sections - Section A - ROC curves to optimise thresholds for correlation score. Section B - Figures S1-S3. Section C - List of ESTs whose identity was inferred by ExprAlign procedure. Click here for file
