# Supplementary material for: ExprAlign - the identification of ESTs in non-model species by alignment of cDNA microarray expression profiles
Source: BMC Genomics. 2009 Nov 26;10:560. doi: 10.1186/1471-2164-10-560 (PMC2790474; doi:10.1186/1471-2164-10-560)
Supplement: Additional file 1 — ExprAlign - the identification of ESTs in non-model species by alignment of cDNA microarray expression profiles. This document contains supplementary material for the abovementioned paper. It includes the following Sections - Section A - ROC curves to optimise thresholds for correlation score. Section B - Figures S1-S3. Section C - List of ESTs whose identity was inferred by ExprAlign procedure. [file 1471-2164-10-560-S1.doc]

# Additional file: ExprAlign – the identification of ESTs in non-model species by alignment of cDNA microarray expression profiles

**Li *et al***

#### Section A: ROC curves to optimise thresholds for correlation scores

A pair of cDNA clones derived from a same gene should share sequence overlap and should in turn poses similar gene expression profiles. Probes constructed from these clones should be clustered together if they are reverse-transcribed from a same region of the gene. In theory their expression correlation coefficient of these two probes should be close to 1. But in practice, gene expression data is noisy and differences in the exact sequence presented in the probe leads to expression differences. This raises the question of how large a correlation score needs to be in order to assign a pair of cDNA clones as copies of the same gene. One task was to define a threshold for correlation scores such that if the correlation for two clones was larger than the threshold, then the two clones were likely to be derived the same gene and that their ESTs should be clustered in a same group in carpBASE 2.1 (http://legr.liv.ac.uk).

The EST clustering as implemented in EST-ferret and in the construction of carpBASE 2.1 was used to define the sequence relatedness of each clone in the dataset. The Relative Operating Characteristic (ROC) was implemented to test the usefulness of the search statistics (<http://www.anaesthetist.com/mnm/stats/roc/>) in order to optimize threshold for correlation scores. The optimized threshold can be obtained by plotting the sensitivity (True Positive, P^+^) of the comparison against the selectivity (False Positive, P^-^) (Anderson and Brass 1998).

**Sensitivity**: P ^+^ = t ^+^ / (t ^+^ + f -) (Equation 4.4)

**Selectivity**: P - = t - / (t - + f ^+^) (Equation 4.5)

where t^+^ is a true positive: two sequences are in a same sub-group (in carpBASE 2.1) and have a gene correlation score above threshold. f^-^ is false negative: two sequences are in a same sub-group but have gene correlation score below threshold; t^-^ is a true negative: two sequences are in different sub-groups and have gene correlation score below threshold; and f^+^ is false positive: two sequences are in different sub-groups but have gene correlation above threshold. If a specific threshold value is defined, it is therefore possible to assign all corrections as true positives, false negatives, true negatives or false positives. The key question is how to select a best threshold for the correlation scores. The criterion is that the best threshold should be able to minimise the total number of errors. The sensitivity P^+^ indicates the probability of the observed true positives at a threshold, so the probability of the missed true positives at a threshold can be given by (1 – P^+^). On the other hand, the selectivity P^-^ shows the probability for the observed true negatives, so the probability of the missed true negatives at a threshold can be given by (1- P^-^). Finally, the total probability of the missing of the true positives and the true negatives can be given by E = (1 - P^+^) + (1 - P^-^). The best threshold should be able to minimise E. A set of thresholds can be tested to calculate the values of P^+^, P^-^ and E. When E is minimised, the optimal threshold is found.

If the transcript expression of two array probes possessed a significant correlation coefficient, we defined these two sequences as a sequence-pair. If sequences in a sequence-pair came from a same sub-group, the sequence-pair was defined as a matched sequence-pair; otherwise, an un-matched sequence-pair. PERL scripts were written to extract the matched sequence-pairs and the un-matched sequence-pairs by examination of their correlation scores. With this information, distributions of t^+^, f^-^, t^-^, and f^+^ were established. With the distributions, the minimum of E could be found and the optimal cut-off could be identified. For example, if the threshold was 0.9, the true positive (t^+^) would be matched sequence-pairs with correlation scores above 0.9; the false negative (f^-^) would be matched sequence-pairs with the correlation scores under 0.9; the false positive (f^+^) would be un-matched sequence-pairs with correlation scores above 0.9; and the true negative (t^-^) would be un-matched sequence-pairs with the correlation scores under 0.9. The curve for E can be illustrated by a line chart in the Microsoft Excel programme.

Section B:

Figure S1: A matrix comparison between the contents of expression landscape features generated using GE and CE data sets. This is the full dataset for Fig 3, and each cell of the matrix includes the number of probes that are common to the indicated GE ad CE features.


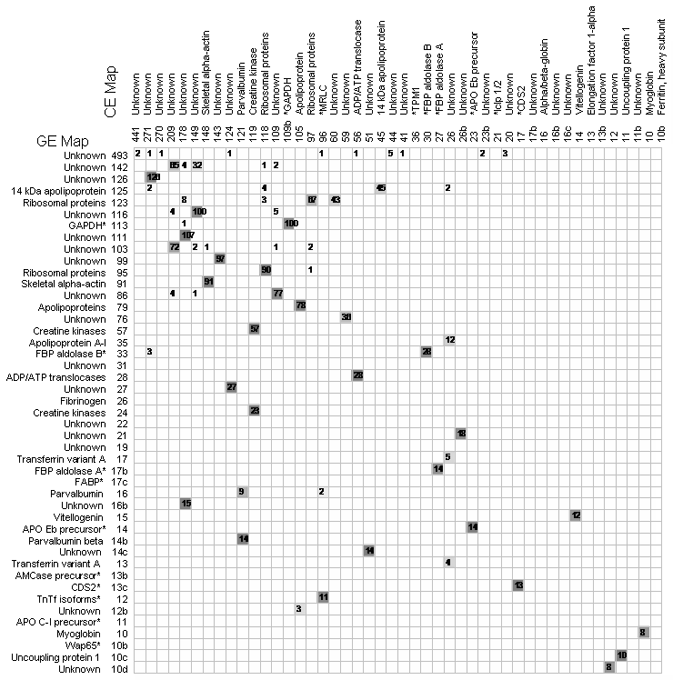


Figure S2: A matrix comparison between the contents of expression landscape features generated using GE and RE data sets. This is the full dataset for Fig 4, and each cell of the matrix includes the number of probes that are common to the indicated GE ad CE features.

**
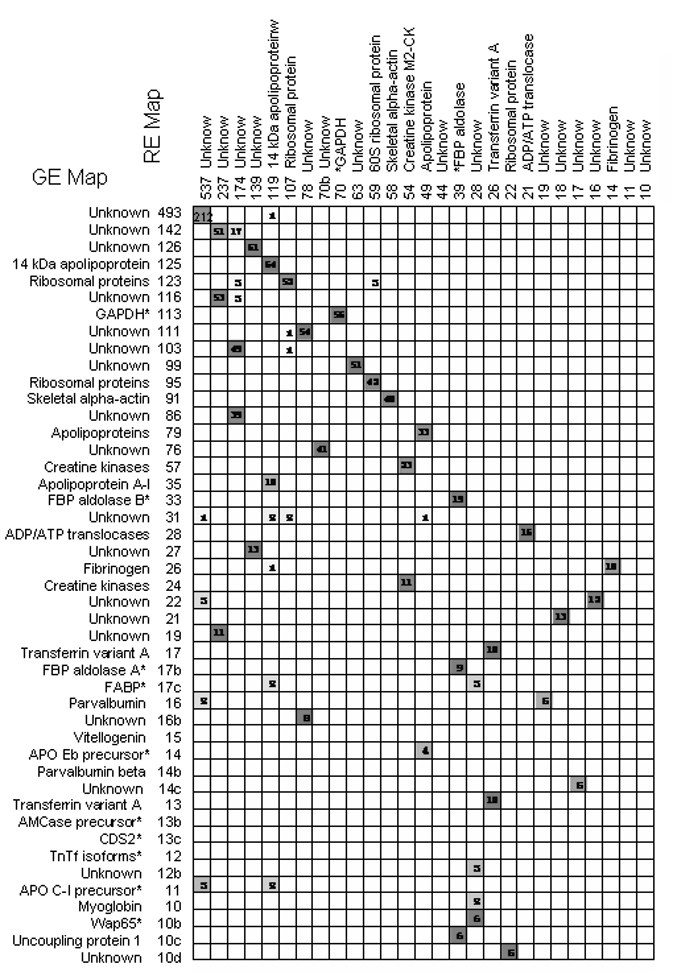
**

**Figure S3:** Heatmaps for other identified mountains in the landscape generated from the GE dataset. Details are as described for Fig 4. Details of the contents of each mountain are included in Table I.


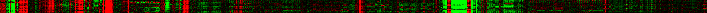

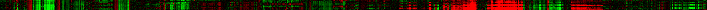

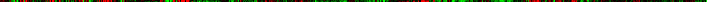

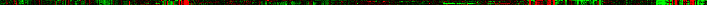

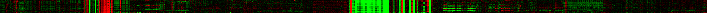

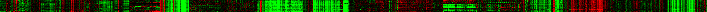

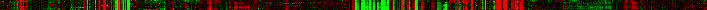

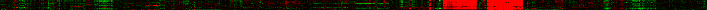

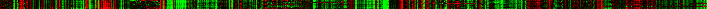

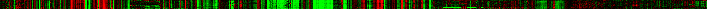

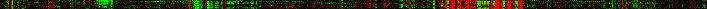

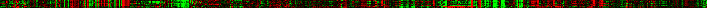

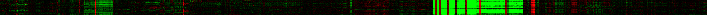

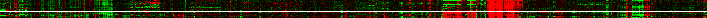

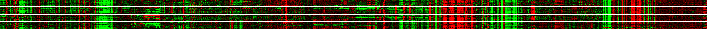

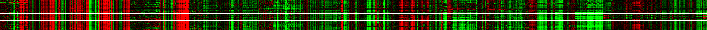

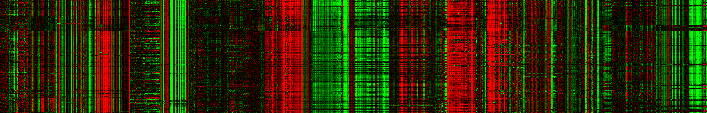

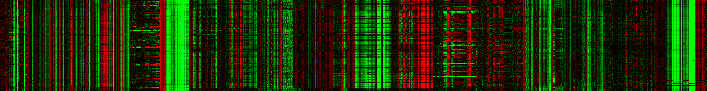


B

**Cold**

G

H

I

K

L

M

B

H

I

M

L

**Hypoxia**

M

L

**Starvation**

17

17

30

30

17

30

17

30

17

30

17c

26

28

91

113

2

5

8

9

9b

9c

10b

10c

12

13b

15

10

13c

**Section C**

**Table S1 – List of ESTs whose identity was inferred by the ExprAlign procedure.**

For all of the clones lacking a Genbank i.d. the sequence was not returned from the high throughput sequencing service provider.

| **Mount-ain** | **carpBASE 2.1**  **i.d.** | **GenBank**  **accession** | **GenBank**  **GI** | **Inferred identity by Expralign** |
| --- | --- | --- | --- | --- |
| GE125 | CC_01A01 | Unsequenced |  | Similar to 14 kDa apolipoprotein [Danio rerio] |
|  | CC_01C02 | Unsequenced |  | Similar to 14 kDa apolipoprotein [Danio rerio] |
|  | CC_01D09 | Unsequenced |  | Similar to 14 kDa apolipoprotein [Danio rerio] |
|  | CC_01K17 | Unsequenced |  | Similar to 14 kDa apolipoprotein [Danio rerio] |
|  | CC_01M19 | Unsequenced |  | Similar to 14 kDa apolipoprotein [Danio rerio] |
|  | CC_01N05 | Unsequenced |  | Similar to 14 kDa apolipoprotein [Danio rerio] |
|  | CC_01O09 | Unsequenced |  | Similar to 14 kDa apolipoprotein [Danio rerio] |
|  | CC_02B18 | Unsequenced |  | Similar to 14 kDa apolipoprotein [Danio rerio] |
|  | CC_02B21 | Unsequenced |  | Similar to 14 kDa apolipoprotein [Danio rerio] |
|  | CC_02C18 | Unsequenced |  | Similar to 14 kDa apolipoprotein [Danio rerio] |
|  | CC_02I24 | Unsequenced |  | Similar to 14 kDa apolipoprotein [Danio rerio] |
|  | CC_02L20 | Unsequenced |  | Similar to 14 kDa apolipoprotein [Danio rerio] |
|  | CC_02N11 | Unsequenced |  | Similar to 14 kDa apolipoprotein [Danio rerio] |
|  | CC_02P04 | Unsequenced |  | Similar to 14 kDa apolipoprotein [Danio rerio] |
|  | CC_03A08 | Unsequenced |  | Similar to 14 kDa apolipoprotein [Danio rerio] |
|  | CC_03C15 | Unsequenced |  | Similar to 14 kDa apolipoprotein [Danio rerio] |
|  | CC_03D04 | Unsequenced |  | Similar to 14 kDa apolipoprotein [Danio rerio] |
|  | CC_03D10 | Unsequenced |  | Similar to 14 kDa apolipoprotein [Danio rerio] |
|  | CC_03D11 | Unsequenced |  | Similar to 14 kDa apolipoprotein [Danio rerio] |
|  | CC_03D12 | Unsequenced |  | Similar to 14 kDa apolipoprotein [Danio rerio] |
|  | CC_03E20 | Unsequenced |  | Similar to 14 kDa apolipoprotein [Danio rerio] |
|  | CC_03F01 | Unsequenced |  | Similar to 14 kDa apolipoprotein [Danio rerio] |
|  | CC_03F07 | Unsequenced |  | Similar to 14 kDa apolipoprotein [Danio rerio] |
|  | CC_03F14 | Unsequenced |  | Similar to 14 kDa apolipoprotein [Danio rerio] |
|  | CC_03G10 | Unsequenced |  | Similar to 14 kDa apolipoprotein [Danio rerio] |
|  | CC_03K09 | Unsequenced |  | Similar to 14 kDa apolipoprotein [Danio rerio] |
|  | CC_03N11 | Unsequenced |  | Similar to 14 kDa apolipoprotein [Danio rerio] |
|  | CC_03N12 | Unsequenced |  | Similar to 14 kDa apolipoprotein [Danio rerio] |
|  | CC_03N22 | Unsequenced |  | Similar to 14 kDa apolipoprotein [Danio rerio] |
|  | CC_03O03 | Unsequenced |  | Similar to 14 kDa apolipoprotein [Danio rerio] |
|  | CC_03O06 | Unsequenced |  | Similar to 14 kDa apolipoprotein [Danio rerio] |
|  | CC_03O07 | Unsequenced |  | Similar to 14 kDa apolipoprotein [Danio rerio] |
|  | CC_04A13 | Unsequenced |  | Similar to 14 kDa apolipoprotein [Danio rerio] |
|  | CC_04B02 | Unsequenced |  | Similar to 14 kDa apolipoprotein [Danio rerio] |
|  | CC_04B14 | Unsequenced |  | Similar to 14 kDa apolipoprotein [Danio rerio] |
|  | CC_04D03 | Unsequenced |  | Similar to 14 kDa apolipoprotein [Danio rerio] |
|  | CC_04E02 | Unsequenced |  | Similar to 14 kDa apolipoprotein [Danio rerio] |
|  | CC_04H23 | Unsequenced |  | Similar to 14 kDa apolipoprotein [Danio rerio] |
|  | CC_04J04 | Unsequenced |  | Similar to 14 kDa apolipoprotein [Danio rerio] |
|  | CC_04L10 | Unsequenced |  | Similar to 14 kDa apolipoprotein [Danio rerio] |
|  | CC_04N05 | Unsequenced |  | Similar to 14 kDa apolipoprotein [Danio rerio] |
|  | CC_04N15 | Unsequenced |  | Similar to 14 kDa apolipoprotein [Danio rerio] |
|  | CC_04N24 | Unsequenced |  | Similar to 14 kDa apolipoprotein [Danio rerio] |
|  | CC_04P05 | Unsequenced |  | Similar to 14 kDa apolipoprotein [Danio rerio] |
|  | CC_04P10 | Unsequenced |  | Similar to 14 kDa apolipoprotein [Danio rerio] |
|  | CC_05A05 | Unsequenced |  | Similar to 14 kDa apolipoprotein [Danio rerio] |
|  | CC_05A07 | Unsequenced |  | Similar to 14 kDa apolipoprotein [Danio rerio] |
|  | CC_05B24 | Unsequenced |  | Similar to 14 kDa apolipoprotein [Danio rerio] |
|  | CC_05C22 | Unsequenced |  | Similar to 14 kDa apolipoprotein [Danio rerio] |
|  | CC_05D24 | Unsequenced |  | Similar to 14 kDa apolipoprotein [Danio rerio] |
|  | CC_05E23 | Unsequenced |  | Similar to 14 kDa apolipoprotein [Danio rerio] |
|  | CC_05J08 | Unsequenced |  | Similar to 14 kDa apolipoprotein [Danio rerio] |
|  | CC_05M08 | Unsequenced |  | Similar to 14 kDa apolipoprotein [Danio rerio] |
|  | CC_05P07 | Unsequenced |  | Similar to 14 kDa apolipoprotein [Danio rerio] |
|  | CC_05P08 | Unsequenced |  | Similar to 14 kDa apolipoprotein [Danio rerio] |
|  | CC_06A06 | Unsequenced |  | Similar to 14 kDa apolipoprotein [Danio rerio] |
|  | CC_06A09 | Unsequenced |  | Similar to 14 kDa apolipoprotein [Danio rerio] |
|  | CC_06C19 | Unsequenced |  | Similar to 14 kDa apolipoprotein [Danio rerio] |
|  | CC_06C20 | Unsequenced |  | Similar to 14 kDa apolipoprotein [Danio rerio] |
|  | CC_06E02 | Unsequenced |  | Similar to 14 kDa apolipoprotein [Danio rerio] |
|  | CC_06E10 | Unsequenced |  | Similar to 14 kDa apolipoprotein [Danio rerio] |
|  | CC_06F22 | Unsequenced |  | Similar to 14 kDa apolipoprotein [Danio rerio] |
|  | CC_06J07 | Unsequenced |  | Similar to 14 kDa apolipoprotein [Danio rerio] |
|  | CC_06J08 | Unsequenced |  | Similar to 14 kDa apolipoprotein [Danio rerio] |
|  | CC_06K22 | Unsequenced |  | Similar to 14 kDa apolipoprotein [Danio rerio] |
|  | CC_06M04 | Unsequenced |  | Similar to 14 kDa apolipoprotein [Danio rerio] |
|  | CC_06M18 | Unsequenced |  | Similar to 14 kDa apolipoprotein [Danio rerio] |
|  | CC_06M21 | Unsequenced |  | Similar to 14 kDa apolipoprotein [Danio rerio] |
|  | CC_06O23 | Unsequenced |  | Similar to 14 kDa apolipoprotein [Danio rerio] |
|  | CC_06P04 | Unsequenced |  | Similar to 14 kDa apolipoprotein [Danio rerio] |
|  | CC_07G01 | Unsequenced |  | Similar to 14 kDa apolipoprotein [Danio rerio] |
|  | CC_07O14 | Unsequenced |  | Similar to 14 kDa apolipoprotein [Danio rerio] |
|  | CC_11A02 | Unsequenced |  | Similar to 14 kDa apolipoprotein [Danio rerio] |
|  | CC_11H19 | Unsequenced |  | Similar to 14 kDa apolipoprotein [Danio rerio] |
|  | CC_35A21 | Unsequenced |  | Similar to 14 kDa apolipoprotein [Danio rerio] |
|  | CC_07I06 | gb|CA964045 | gi|50737019 | Similar to 14 kDa apolipoprotein [Danio rerio] |
|  | CC_05B23 | gb|CA965380 | gi|50738748 | Similar to 14 kDa apolipoprotein [Danio rerio] |
| GE123 | CC_01A06 | Unsequenced |  | Ribosomal proteins |
|  | CC_01C03 | Unsequenced |  | Ribosomal proteins |
|  | CC_01E21 | Unsequenced |  | Ribosomal proteins |
|  | CC_01I14 | Unsequenced |  | Ribosomal proteins |
|  | CC_01J10 | Unsequenced |  | Ribosomal proteins |
|  | CC_01L18 | Unsequenced |  | Ribosomal proteins |
|  | CC_01O15 | Unsequenced |  | Ribosomal proteins |
|  | CC_02F05 | Unsequenced |  | Ribosomal proteins |
|  | CC_02F20 | Unsequenced |  | Ribosomal proteins |
|  | CC_02H15 | Unsequenced |  | Ribosomal proteins |
|  | CC_02L04 | Unsequenced |  | Ribosomal proteins |
|  | CC_02L15 | Unsequenced |  | Ribosomal proteins |
|  | CC_02P05 | Unsequenced |  | Ribosomal proteins |
|  | CC_02P18 | Unsequenced |  | Ribosomal proteins |
|  | CC_03D08 | Unsequenced |  | Ribosomal proteins |
|  | CC_04A06 | Unsequenced |  | Ribosomal proteins |
|  | CC_04E01 | Unsequenced |  | Ribosomal proteins |
|  | CC_04F17 | Unsequenced |  | Ribosomal proteins |
|  | CC_04F23 | Unsequenced |  | Ribosomal proteins |
|  | CC_04L02 | Unsequenced |  | Ribosomal proteins |
|  | CC_04M14 | Unsequenced |  | Ribosomal proteins |
|  | CC_04N20 | Unsequenced |  | Ribosomal proteins |
|  | CC_05J05 | Unsequenced |  | Ribosomal proteins |
|  | CC_07F23 | Unsequenced |  | Ribosomal proteins |
|  | CC_11A24 | Unsequenced |  | Ribosomal proteins |
|  | CC_11B23 | Unsequenced |  | Ribosomal proteins |
|  | CC_11G12 | Unsequenced |  | Ribosomal proteins |
|  | CC_11G19 | Unsequenced |  | Ribosomal proteins |
|  | CC_11J01 | Unsequenced |  | Ribosomal proteins |
|  | CC_11K16 | Unsequenced |  | Ribosomal proteins |
|  | CC_11M24 | Unsequenced |  | Ribosomal proteins |
|  | CC_11P05 | Unsequenced |  | Ribosomal proteins |
|  | CC_12D18 | Unsequenced |  | Ribosomal proteins |
|  | CC_12D22 | Unsequenced |  | Ribosomal proteins |
|  | CC_12F08 | Unsequenced |  | Ribosomal proteins |
|  | CC_12L02 | Unsequenced |  | Ribosomal proteins |
|  | CC_21F20 | Unsequenced |  | Ribosomal proteins |
|  | CC_21I12 | Unsequenced |  | Ribosomal proteins |
|  | CC_25K02 | Unsequenced |  | Ribosomal proteins |
|  | CC_26B01 | Unsequenced |  | Ribosomal proteins |
|  | CC_26D03 | Unsequenced |  | Ribosomal proteins |
|  | CC_26F19 | Unsequenced |  | Ribosomal proteins |
|  | CC_26G20 | Unsequenced |  | Ribosomal proteins |
|  | CC_26H04 | Unsequenced |  | Ribosomal proteins |
|  | CC_26P18 | Unsequenced |  | Ribosomal proteins |
|  | CC_27F20 | Unsequenced |  | Ribosomal proteins |
|  | CC_27F24 | Unsequenced |  | Ribosomal proteins |
|  | CC_28E17 | Unsequenced |  | Ribosomal proteins |
|  | CC_28I19 | Unsequenced |  | Ribosomal proteins |
|  | CC_28O13 | Unsequenced |  | Ribosomal proteins |
|  | CC_29E15 | Unsequenced |  | Ribosomal proteins |
|  | CC_29L24 | Unsequenced |  | Ribosomal proteins |
|  | CC_30J05 | Unsequenced |  | Ribosomal proteins |
|  | CC_34A14 | Unsequenced |  | Ribosomal proteins |
|  | CC_34E11 | Unsequenced |  | Ribosomal proteins |
|  | CC_35A13 | Unsequenced |  | Ribosomal proteins |
|  | CC_35L08 | Unsequenced |  | Ribosomal proteins |
|  | CC_02G08 | gb|CA967380 | gi|50733213 | Ribosomal proteins |
|  | CC_07G17 | gb|CA964034 | gi|50737008 | Ribosomal proteins |
|  | CC_12C17 | gb|CA967743 | gi|50733789 | Ribosomal proteins |
|  | CC_12E08 | gb|CA970239 | gi|50736745 | Ribosomal proteins |
|  | CC_12F14 | gb|CA966347 | gi|50739830 | Ribosomal proteins |
|  | CC_12I19 | gb|CA968925 | gi|50735148 | Ribosomal proteins |
|  | CC_12J08 | gb|CA967118 | gi|50740954 | Ribosomal proteins |
|  | CC_21E03 | gb|CA967913 | gi|50733956 | Ribosomal proteins |
|  | CC_27C24 | gb|CA968241 | gi|50734391 | Ribosomal proteins |
|  | CC_33L16 | gb|CF661082 | gi|50743279 | Ribosomal proteins |
|  | CC_33G21 | gb|CF660993 | gi|50743183 | Ribosomal proteins |
|  | CC_35L21 | gb|CF661566 | gi|50743824 | Ribosomal proteins |
|  | CC_35F03 | gb|CF661467 | gi|50743708 | Ribosomal proteins |
| GE113 | CC_01E19 | Unsequenced |  | Glyceraldehyde-3-phosphate dehydrogenase |
|  | CC_13B06 | Unsequenced |  | Glyceraldehyde-3-phosphate dehydrogenase |
|  | CC_05H21 | Unsequenced |  | Glyceraldehyde-3-phosphate dehydrogenase |
|  | CC_06A16 | Unsequenced |  | Glyceraldehyde-3-phosphate dehydrogenase |
|  | CC_06E23 | Unsequenced |  | Glyceraldehyde-3-phosphate dehydrogenase |
|  | CC_06I16 | Unsequenced |  | Glyceraldehyde-3-phosphate dehydrogenase |
|  | CC_07N13 | Unsequenced |  | Glyceraldehyde-3-phosphate dehydrogenase |
|  | CC_13F20 | Unsequenced |  | Glyceraldehyde-3-phosphate dehydrogenase |
|  | CC_13N19 | Unsequenced |  | Glyceraldehyde-3-phosphate dehydrogenase |
|  | CC_13P18 | Unsequenced |  | Glyceraldehyde-3-phosphate dehydrogenase |
|  | CC_13J14 | Unsequenced |  | Glyceraldehyde-3-phosphate dehydrogenase |
|  | CC_13M12 | Unsequenced |  | Glyceraldehyde-3-phosphate dehydrogenase |
|  | CC_26A13 | Unsequenced |  | Glyceraldehyde-3-phosphate dehydrogenase |
|  | CC_26A21 | Unsequenced |  | Glyceraldehyde-3-phosphate dehydrogenase |
|  | CC_26N07 | Unsequenced |  | Glyceraldehyde-3-phosphate dehydrogenase |
|  | CC_26N14 | Unsequenced |  | Glyceraldehyde-3-phosphate dehydrogenase |
|  | CC_26P24 | Unsequenced |  | Glyceraldehyde-3-phosphate dehydrogenase |
|  | CC_26D19 | Unsequenced |  | Glyceraldehyde-3-phosphate dehydrogenase |
|  | CC_26J20 | Unsequenced |  | Glyceraldehyde-3-phosphate dehydrogenase |
|  | CC_26K23 | Unsequenced |  | Glyceraldehyde-3-phosphate dehydrogenase |
|  | CC_27C07 | Unsequenced |  | Glyceraldehyde-3-phosphate dehydrogenase |
|  | CC_27L12 | Unsequenced |  | Glyceraldehyde-3-phosphate dehydrogenase |
|  | CC_27O21 | Unsequenced |  | Glyceraldehyde-3-phosphate dehydrogenase |
|  | CC_27J19 | Unsequenced |  | Glyceraldehyde-3-phosphate dehydrogenase |
|  | CC_27P14 | Unsequenced |  | Glyceraldehyde-3-phosphate dehydrogenase |
|  | CC_28A05 | Unsequenced |  | Glyceraldehyde-3-phosphate dehydrogenase |
|  | CC_28A06 | Unsequenced |  | Glyceraldehyde-3-phosphate dehydrogenase |
|  | CC_28F11 | Unsequenced |  | Glyceraldehyde-3-phosphate dehydrogenase |
|  | CC_28F13 | Unsequenced |  | Glyceraldehyde-3-phosphate dehydrogenase |
|  | CC_28G04 | Unsequenced |  | Glyceraldehyde-3-phosphate dehydrogenase |
|  | CC_28J24 | Unsequenced |  | Glyceraldehyde-3-phosphate dehydrogenase |
|  | CC_29C17 | Unsequenced |  | Glyceraldehyde-3-phosphate dehydrogenase |
|  | CC_29C21 | Unsequenced |  | Glyceraldehyde-3-phosphate dehydrogenase |
|  | CC_29D13 | Unsequenced |  | Glyceraldehyde-3-phosphate dehydrogenase |
|  | CC_29D20 | Unsequenced |  | Glyceraldehyde-3-phosphate dehydrogenase |
|  | CC_29E05 | Unsequenced |  | Glyceraldehyde-3-phosphate dehydrogenase |
|  | CC_29L03 | Unsequenced |  | Glyceraldehyde-3-phosphate dehydrogenase |
|  | CC_28C21 | Unsequenced |  | Glyceraldehyde-3-phosphate dehydrogenase |
|  | CC_28D05 | Unsequenced |  | Glyceraldehyde-3-phosphate dehydrogenase |
|  | CC_28H21 | Unsequenced |  | Glyceraldehyde-3-phosphate dehydrogenase |
|  | CC_28L20 | Unsequenced |  | Glyceraldehyde-3-phosphate dehydrogenase |
|  | CC_28M15 | Unsequenced |  | Glyceraldehyde-3-phosphate dehydrogenase |
|  | CC_29B03 | Unsequenced |  | Glyceraldehyde-3-phosphate dehydrogenase |
|  | CC_29B23 | Unsequenced |  | Glyceraldehyde-3-phosphate dehydrogenase |
|  | CC_29C11 | Unsequenced |  | Glyceraldehyde-3-phosphate dehydrogenase |
|  | CC_29C16 | Unsequenced |  | Glyceraldehyde-3-phosphate dehydrogenase |
|  | CC_29H09 | Unsequenced |  | Glyceraldehyde-3-phosphate dehydrogenase |
|  | CC_29H13 | Unsequenced |  | Glyceraldehyde-3-phosphate dehydrogenase |
|  | CC_29L18 | Unsequenced |  | Glyceraldehyde-3-phosphate dehydrogenase |
|  | CC_29N04 | Unsequenced |  | Glyceraldehyde-3-phosphate dehydrogenase |
|  | CC_29N05 | Unsequenced |  | Glyceraldehyde-3-phosphate dehydrogenase |
|  | CC_29N21 | Unsequenced |  | Glyceraldehyde-3-phosphate dehydrogenase |
|  | CC_29O24 | Unsequenced |  | Glyceraldehyde-3-phosphate dehydrogenase |
|  | CC_30K13 | Unsequenced |  | Glyceraldehyde-3-phosphate dehydrogenase |
|  | CC_30G19 | Unsequenced |  | Glyceraldehyde-3-phosphate dehydrogenase |
|  | CC_30G23 | Unsequenced |  | Glyceraldehyde-3-phosphate dehydrogenase |
|  | CC_30L14 | Unsequenced |  | Glyceraldehyde-3-phosphate dehydrogenase |
|  | CC_30P07 | Unsequenced |  | Glyceraldehyde-3-phosphate dehydrogenase |
| GE95 | CC_01M06 | Unsequenced |  | Ribosomal proteins |
|  | CC_03A11 | Unsequenced |  | Ribosomal proteins |
|  | CC_03A12 | Unsequenced |  | Ribosomal proteins |
|  | CC_03A24 | Unsequenced |  | Ribosomal proteins |
|  | CC_03B11 | Unsequenced |  | Ribosomal proteins |
|  | CC_03B12 | Unsequenced |  | Ribosomal proteins |
|  | CC_03B13 | Unsequenced |  | Ribosomal proteins |
|  | CC_03B23 | Unsequenced |  | Ribosomal proteins |
|  | CC_03C20 | Unsequenced |  | Ribosomal proteins |
|  | CC_03E08 | Unsequenced |  | Ribosomal proteins |
|  | CC_03E09 | Unsequenced |  | Ribosomal proteins |
|  | CC_03E10 | Unsequenced |  | Ribosomal proteins |
|  | CC_03F02 | Unsequenced |  | Ribosomal proteins |
|  | CC_03F12 | Unsequenced |  | Ribosomal proteins |
|  | CC_03F19 | Unsequenced |  | Ribosomal proteins |
|  | CC_03G12 | Unsequenced |  | Ribosomal proteins |
|  | CC_03G13 | Unsequenced |  | Ribosomal proteins |
|  | CC_03G14 | Unsequenced |  | Ribosomal proteins |
|  | CC_03G24 | Unsequenced |  | Ribosomal proteins |
|  | CC_03H02 | Unsequenced |  | Ribosomal proteins |
|  | CC_03H07 | Unsequenced |  | Ribosomal proteins |
|  | CC_03H10 | Unsequenced |  | Ribosomal proteins |
|  | CC_03H12 | Unsequenced |  | Ribosomal proteins |
|  | CC_03H13 | Unsequenced |  | Ribosomal proteins |
|  | CC_03H14 | Unsequenced |  | Ribosomal proteins |
|  | CC_03H18 | Unsequenced |  | Ribosomal proteins |
|  | CC_03I12 | Unsequenced |  | Ribosomal proteins |
|  | CC_03I13 | Unsequenced |  | Ribosomal proteins |
|  | CC_03J02 | Unsequenced |  | Ribosomal proteins |
|  | CC_03J12 | Unsequenced |  | Ribosomal proteins |
|  | CC_03J14 | Unsequenced |  | Ribosomal proteins |
|  | CC_03J24 | Unsequenced |  | Ribosomal proteins |
|  | CC_03K13 | Unsequenced |  | Ribosomal proteins |
|  | CC_03K14 | Unsequenced |  | Ribosomal proteins |
|  | CC_03K18 | Unsequenced |  | Ribosomal proteins |
|  | CC_03L11 | Unsequenced |  | Ribosomal proteins |
|  | CC_03L14 | Unsequenced |  | Ribosomal proteins |
|  | CC_03L23 | Unsequenced |  | Ribosomal proteins |
|  | CC_03M01 | Unsequenced |  | Ribosomal proteins |
|  | CC_03M05 | Unsequenced |  | Ribosomal proteins |
|  | CC_03M13 | Unsequenced |  | Ribosomal proteins |
|  | CC_03M14 | Unsequenced |  | Ribosomal proteins |
|  | CC_03M17 | Unsequenced |  | Ribosomal proteins |
|  | CC_03N01 | Unsequenced |  | Ribosomal proteins |
|  | CC_03N07 | Unsequenced |  | Ribosomal proteins |
|  | CC_03O08 | Unsequenced |  | Ribosomal proteins |
|  | CC_03O09 | Unsequenced |  | Ribosomal proteins |
|  | CC_03O10 | Unsequenced |  | Ribosomal proteins |
|  | CC_03O12 | Unsequenced |  | Ribosomal proteins |
|  | CC_03O13 | Unsequenced |  | Ribosomal proteins |
|  | CC_03O16 | Unsequenced |  | Ribosomal proteins |
|  | CC_03P03 | Unsequenced |  | Ribosomal proteins |
|  | CC_03P04 | Unsequenced |  | Ribosomal proteins |
|  | CC_03P07 | Unsequenced |  | Ribosomal proteins |
|  | CC_03P08 | Unsequenced |  | Ribosomal proteins |
|  | CC_03P09 | Unsequenced |  | Ribosomal proteins |
|  | CC_03P11 | Unsequenced |  | Ribosomal proteins |
|  | CC_03P12 | Unsequenced |  | Ribosomal proteins |
|  | CC_03P13 | Unsequenced |  | Ribosomal proteins |
|  | CC_03P16 | Unsequenced |  | Ribosomal proteins |
|  | CC_03P19 | Unsequenced |  | Ribosomal proteins |
|  | CC_03P20 | Unsequenced |  | Ribosomal proteins |
|  | CC_03P23 | Unsequenced |  | Ribosomal proteins |
|  | CC_03P24 | Unsequenced |  | Ribosomal proteins |
|  | CC_04M20 | Unsequenced |  | Ribosomal proteins |
|  | CC_11G06 | Unsequenced |  | Ribosomal proteins |
|  | CC_12B10 | Unsequenced |  | Ribosomal proteins |
|  | CC_12J06 | Unsequenced |  | Ribosomal proteins |
|  | CC_12L16 | Unsequenced |  | Ribosomal proteins |
|  | CC_16E24 | Unsequenced |  | Ribosomal proteins |
|  | CC_40L23 | Unsequenced |  | Ribosomal proteins |
|  | CC_03A20 | gb|CA966869 | gi|50740624 | Ribosomal proteins |
| GE91 | CC_04G22 | Unsequenced |  | Skeletal alpha-actin |
|  | CC_05D05 | Unsequenced |  | Skeletal alpha-actin |
|  | CC_06B14 | Unsequenced |  | Skeletal alpha-actin |
|  | CC_06F16 | Unsequenced |  | Skeletal alpha-actin |
|  | CC_06J12 | Unsequenced |  | Skeletal alpha-actin |
|  | CC_06P12 | Unsequenced |  | Skeletal alpha-actin |
|  | CC_21B20 | Unsequenced |  | Skeletal alpha-actin |
|  | CC_21B24 | Unsequenced |  | Skeletal alpha-actin |
|  | CC_21D10 | Unsequenced |  | Skeletal alpha-actin |
|  | CC_21K22 | Unsequenced |  | Skeletal alpha-actin |
|  | CC_21L10 | Unsequenced |  | Skeletal alpha-actin |
|  | CC_21P03 | Unsequenced |  | Skeletal alpha-actin |
|  | CC_22C23 | Unsequenced |  | Skeletal alpha-actin |
|  | CC_22J07 | Unsequenced |  | Skeletal alpha-actin |
|  | CC_22P12 | Unsequenced |  | Skeletal alpha-actin |
|  | CC_24F16 | Unsequenced |  | Skeletal alpha-actin |
|  | CC_25M06 | Unsequenced |  | Skeletal alpha-actin |
|  | CC_25M17 | Unsequenced |  | Skeletal alpha-actin |
|  | CC_25M18 | Unsequenced |  | Skeletal alpha-actin |
|  | CC_26L04 | Unsequenced |  | Skeletal alpha-actin |
|  | CC_26L07 | Unsequenced |  | Skeletal alpha-actin |
|  | CC_26N13 | Unsequenced |  | Skeletal alpha-actin |
|  | CC_28A13 | Unsequenced |  | Skeletal alpha-actin |
|  | CC_28A17 | Unsequenced |  | Skeletal alpha-actin |
|  | CC_28K07 | Unsequenced |  | Skeletal alpha-actin |
|  | CC_28O15 | Unsequenced |  | Skeletal alpha-actin |
|  | CC_28O16 | Unsequenced |  | Skeletal alpha-actin |
|  | CC_28P16 | Unsequenced |  | Skeletal alpha-actin |
|  | CC_29A05 | Unsequenced |  | Skeletal alpha-actin |
|  | CC_29B01 | Unsequenced |  | Skeletal alpha-actin |
|  | CC_29C24 | Unsequenced |  | Skeletal alpha-actin |
|  | CC_29E07 | Unsequenced |  | Skeletal alpha-actin |
|  | CC_29E12 | Unsequenced |  | Skeletal alpha-actin |
|  | CC_29F15 | Unsequenced |  | Skeletal alpha-actin |
|  | CC_29F17 | Unsequenced |  | Skeletal alpha-actin |
|  | CC_29J15 | Unsequenced |  | Skeletal alpha-actin |
|  | CC_29L01 | Unsequenced |  | Skeletal alpha-actin |
|  | CC_29P19 | Unsequenced |  | Skeletal alpha-actin |
|  | CC_21H23 | gb|CA969193 | gi|50735415 | Skeletal alpha-actin |
|  | CC_22A23 | gb|CA969239 | gi|50735461 | Skeletal alpha-actin |
|  | CC_22K23 | gb|CA969439 | gi|50735661 | Skeletal alpha-actin |
|  | CC_24O04 | gb|CA969990 | gi|50736441 | Skeletal alpha-actin |
|  | CC_26M22 | gb|CA970361 | gi|50736882 | Skeletal alpha-actin |
|  | CC_25F24 | gb|CF662928 | gi|50742057 | Skeletal alpha-actin |
|  | CC_32D23 | gb|CF660675 | gi|50742801 | Skeletal alpha-actin |
| GE79 | CC_01A17 | Unsequenced |  | Apolipoproteins |
|  | CC_01F15 | Unsequenced |  | Apolipoproteins |
|  | CC_01I17 | Unsequenced |  | Apolipoproteins |
|  | CC_01L12 | Unsequenced |  | Apolipoproteins |
|  | CC_01O18 | Unsequenced |  | Apolipoproteins |
|  | CC_02B02 | Unsequenced |  | Apolipoproteins |
|  | CC_02J17 | Unsequenced |  | Apolipoproteins |
|  | CC_02N01 | Unsequenced |  | Apolipoproteins |
|  | CC_02N19 | Unsequenced |  | Apolipoproteins |
|  | CC_03F18 | Unsequenced |  | Apolipoproteins |
|  | CC_04B13 | Unsequenced |  | Apolipoproteins |
|  | CC_04H18 | Unsequenced |  | Apolipoproteins |
|  | CC_04J02 | Unsequenced |  | Apolipoproteins |
|  | CC_04L03 | Unsequenced |  | Apolipoproteins |
|  | CC_04L11 | Unsequenced |  | Apolipoproteins |
|  | CC_05F22 | Unsequenced |  | Apolipoproteins |
|  | CC_05H05 | Unsequenced |  | Apolipoproteins |
|  | CC_05N12 | Unsequenced |  | Apolipoproteins |
|  | CC_06F18 | Unsequenced |  | Apolipoproteins |
|  | CC_06O03 | Unsequenced |  | Apolipoproteins |
|  | CC_06O22 | Unsequenced |  | Apolipoproteins |
|  | CC_07F22 | Unsequenced |  | Apolipoproteins |
|  | CC_07P08 | Unsequenced |  | Apolipoproteins |
|  | CC_11H13 | Unsequenced |  | Apolipoproteins |
|  | CC_12P16 | Unsequenced |  | Apolipoproteins |
|  | CC_13C10 | Unsequenced |  | Apolipoproteins |
|  | CC_13D20 | Unsequenced |  | Apolipoproteins |
|  | CC_13N07 | Unsequenced |  | Apolipoproteins |
|  | CC_15J19 | Unsequenced |  | Apolipoproteins |
|  | CC_16B21 | Unsequenced |  | Apolipoproteins |
|  | CC_16F19 | Unsequenced |  | Apolipoproteins |
|  | CC_16G23 | Unsequenced |  | Apolipoproteins |
|  | CC_24J01 | Unsequenced |  | Apolipoproteins |
|  | CC_26D21 | Unsequenced |  | Apolipoproteins |
|  | CC_26J08 | Unsequenced |  | Apolipoproteins |
|  | CC_27B04 | Unsequenced |  | Apolipoproteins |
|  | CC_29I01 | Unsequenced |  | Apolipoproteins |
|  | CC_12O16 | gb|CA970291 | gi|50736796 | Apolipoproteins |
|  | CC_24J09 | gb|CA969897 | gi|50736333 | Apolipoproteins |
|  | CC_28A03 | gb|CA966582 | gi|50740067 | Apolipoproteins |
| GE57 | CC_13B16 | Unsequenced |  | Creatine kinases |
|  | CC_13G02 | Unsequenced |  | Creatine kinases |
|  | CC_14C10 | Unsequenced |  | Creatine kinases |
|  | CC_21G14 | Unsequenced |  | Creatine kinases |
|  | CC_25L22 | Unsequenced |  | Creatine kinases |
|  | CC_26C20 | Unsequenced |  | Creatine kinases |
|  | CC_26D20 | Unsequenced |  | Creatine kinases |
|  | CC_26E06 | Unsequenced |  | Creatine kinases |
|  | CC_26E22 | Unsequenced |  | Creatine kinases |
|  | CC_26H20 | Unsequenced |  | Creatine kinases |
|  | CC_26L21 | Unsequenced |  | Creatine kinases |
|  | CC_26N17 | Unsequenced |  | Creatine kinases |
|  | CC_27H20 | Unsequenced |  | Creatine kinases |
|  | CC_27N14 | Unsequenced |  | Creatine kinases |
|  | CC_28F06 | Unsequenced |  | Creatine kinases |
|  | CC_28F21 | Unsequenced |  | Creatine kinases |
|  | CC_28K09 | Unsequenced |  | Creatine kinases |
|  | CC_28M19 | Unsequenced |  | Creatine kinases |
|  | CC_28O03 | Unsequenced |  | Creatine kinases |
|  | CC_28P11 | Unsequenced |  | Creatine kinases |
|  | CC_29D02 | Unsequenced |  | Creatine kinases |
|  | CC_29F23 | Unsequenced |  | Creatine kinases |
|  | CC_29F24 | Unsequenced |  | Creatine kinases |
|  | CC_29I15 | Unsequenced |  | Creatine kinases |
|  | CC_29K03 | Unsequenced |  | Creatine kinases |
|  | CC_29O04 | Unsequenced |  | Creatine kinases |
|  | CC_30C13 | Unsequenced |  | Creatine kinases |
|  | CC_34C09 | Unsequenced |  | Creatine kinases |
|  | CC_34E22 | Unsequenced |  | Creatine kinases |
|  | CC_13N24 | gb|CA966395 | gi|50739878 | Creatine kinases |
|  | CC_32D22 | gb|CF660674 | gi|50742800 | Creatine kinases |
|  | CC_32E02 | gb|CF660678 | gi|50742804 | Creatine kinases |
|  | CC_32F01 | gb|CF660692 | gi|50742820 | Creatine kinases |
| GE35 | CC_01G08 | Unsequenced |  | Apolipoprotein A-I |
|  | CC_01K09 | Unsequenced |  | Apolipoprotein A-I |
|  | CC_01L11 | Unsequenced |  | Apolipoprotein A-I |
|  | CC_01L15 | Unsequenced |  | Apolipoprotein A-I |
|  | CC_01M09 | Unsequenced |  | Apolipoprotein A-I |
|  | CC_02B04 | Unsequenced |  | Apolipoprotein A-I |
|  | CC_02B15 | Unsequenced |  | Apolipoprotein A-I |
|  | CC_02D12 | Unsequenced |  | Apolipoprotein A-I |
|  | CC_02E20 | Unsequenced |  | Apolipoprotein A-I |
|  | CC_02H03 | Unsequenced |  | Apolipoprotein A-I |
|  | CC_02J14 | Unsequenced |  | Apolipoprotein A-I |
|  | CC_02K05 | Unsequenced |  | Apolipoprotein A-I |
|  | CC_03D16 | Unsequenced |  | Apolipoprotein A-I |
|  | CC_04D15 | Unsequenced |  | Apolipoprotein A-I |
|  | CC_04E21 | Unsequenced |  | Apolipoprotein A-I |
|  | CC_04F05 | Unsequenced |  | Apolipoprotein A-I |
|  | CC_04F14 | Unsequenced |  | Apolipoprotein A-I |
|  | CC_04O12 | Unsequenced |  | Apolipoprotein A-I |
|  | CC_05B08 | Unsequenced |  | Apolipoprotein A-I |
|  | CC_07E18 | Unsequenced |  | Apolipoprotein A-I |
| GE33 | CC_05M10 | Unsequenced |  | Fructose-bisphosphate aldolase B |
|  | CC_07J02 | Unsequenced |  | Fructose-bisphosphate aldolase B |
|  | CC_04C07 | gb|CA965177 | gi|50738427 | Fructose-bisphosphate aldolase B |
|  | CC_05G04 | gb|CA965511 | gi|50738879 | Fructose-bisphosphate aldolase B |
|  | CC_07M11 | gb|CF662554 | gi|50741550 | Fructose-bisphosphate aldolase B |
| GE28 | CC_05J11 | Unsequenced |  | ADP/ATP translocases |
|  | CC_07K01 | gb|CF662551 | gi|50741547 | ADP/ATP translocases |
| GE26 | CC_01F21 | Unsequenced |  | Fibrinogen |
|  | CC_01J21 | Unsequenced |  | Fibrinogen |
|  | CC_01K05 | Unsequenced |  | Fibrinogen |
|  | CC_02D22 | Unsequenced |  | Fibrinogen |
|  | CC_06A13 | Unsequenced |  | Fibrinogen |
|  | CC_06B20 | Unsequenced |  | Fibrinogen |
|  | CC_06J22 | Unsequenced |  | Fibrinogen |
|  | CC_07O20 | Unsequenced |  | Fibrinogen |
|  | CC_07E07 | gb|CA964011 | gi|50736985 | Fibrinogen |
|  | CC_07D09 | gb|CA968475 | gi|50734642 | Fibrinogen |
| GE24 | CC_24D02 | Unsequenced |  | Creatine kinases |
|  | CC_26J23 | Unsequenced |  | Creatine kinases |
|  | CC_27E17 | Unsequenced |  | Creatine kinases |
|  | CC_27G16 | Unsequenced |  | Creatine kinases |
|  | CC_28D12 | Unsequenced |  | Creatine kinases |
|  | CC_28J23 | Unsequenced |  | Creatine kinases |
|  | CC_28M21 | Unsequenced |  | Creatine kinases |
|  | CC_28M22 | Unsequenced |  | Creatine kinases |
|  | CC_29C08 | Unsequenced |  | Creatine kinases |
|  | CC_29E02 | Unsequenced |  | Creatine kinases |
|  | CC_29N11 | Unsequenced |  | Creatine kinases |
|  | CC_29O02 | Unsequenced |  | Creatine kinases |
|  | CC_34D03 | Unsequenced |  | Creatine kinases |
|  | CC_34G19 | Unsequenced |  | Creatine kinases |
|  | CC_34M05 | Unsequenced |  | Creatine kinases |
|  | CC_34N19 | Unsequenced |  | Creatine kinases |
|  | CC_34O03 | Unsequenced |  | Creatine kinases |
|  | CC_27I14 | gb|CA968315 | gi|50734465 | Creatine kinases |
| GE17 | CC_01G04 | Unsequenced |  | Transferrin variant A |
|  | CC_01H14 | Unsequenced |  | Transferrin variant A |
|  | CC_01P18 | Unsequenced |  | Transferrin variant A |
|  | CC_03B20 | Unsequenced |  | Transferrin variant A |
|  | CC_04A22 | Unsequenced |  | Transferrin variant A |
|  | CC_04O11 | Unsequenced |  | Transferrin variant A |
|  | CC_06C16 | Unsequenced |  | Transferrin variant A |
|  | CC_06J03 | Unsequenced |  | Transferrin variant A |
| GE17b | CC_24J22 | gb|CA969909 | gi|50736359 | Fructose-bisphosphate aldolase A |
|  | CC_32P02 | Unsequenced |  | Fructose-bisphosphate aldolase A |
| GE17c | CC_08A18 | Unsequenced |  | Fatty acid-binding protein |
|  | CC_11L07 | Unsequenced |  | Fatty acid-binding protein |
|  | CC_10B02 | gb|CA964179 | gi|50737232 | Fatty acid-binding protein |
|  | CC_39I17 | gb|CF661802 | gi|50744076 | Fatty acid-binding protein |
| GE16 | CC_16P22 | Unsequenced |  | Parvalbumins |
|  | CC_20H12 | Unsequenced |  | Parvalbumins |
|  | CC_21E10 | Unsequenced |  | Parvalbumins |
|  | CC_21O24 | Unsequenced |  | Parvalbumins |
|  | CC_26B20 | Unsequenced |  | Parvalbumins |
|  | CC_26O19 | Unsequenced |  | Parvalbumins |
|  | CC_27A21 | Unsequenced |  | Parvalbumins |
| GE15 | CC_01N19 | Unsequenced |  | Vitellogenin |
|  | CC_01P13 | Unsequenced |  | Vitellogenin |
|  | CC_03A17 | Unsequenced |  | Vitellogenin |
|  | CC_03C14 | Unsequenced |  | Vitellogenin |
|  | CC_04D14 | Unsequenced |  | Vitellogenin |
|  | CC_04N03 | Unsequenced |  | Vitellogenin |
|  | CC_06L21 | Unsequenced |  | Vitellogenin |
|  | CC_07A05 | Unsequenced |  | Vitellogenin |
|  | CC_07A15 | Unsequenced |  | Vitellogenin |
| GE14 | CC_01I18 | Unsequenced |  | Apolipoprotein Eb precursor |
|  | CC_05N19 | Unsequenced |  | Apolipoprotein Eb precursor |
|  | CC_06J18 | Unsequenced |  | Apolipoprotein Eb precursor |
|  | CC_07P16 | Unsequenced |  | Apolipoprotein Eb precursor |
|  | CC_29I08 | Unsequenced |  | Apolipoprotein Eb precursor |
|  | CC_35E01 | Unsequenced |  | Apolipoprotein Eb precursor |
|  | CC_35I09 | Unsequenced |  | Apolipoprotein Eb precursor |
|  | CC_35M07 | Unsequenced |  | Apolipoprotein Eb precursor |
|  | CC_07D13 | gb|CA968478 | gi|50734645 | Apolipoprotein Eb precursor |
| GE13 | CC_03A19 | Unsequenced |  | Transferrin variant A |
|  | CC_03I18 | Unsequenced |  | Transferrin variant A |
|  | CC_03N19 | Unsequenced |  | Transferrin variant A |
|  | CC_07H18 | Unsequenced |  | Transferrin variant A |
| GE13b | CC_15G15 | Unsequenced |  | Acidic mammalian chitinase precursor |
|  | CC_16O12 | Unsequenced |  | Acidic mammalian chitinase precursor |
|  | CC_17G13 | Unsequenced |  | Acidic mammalian chitinase precursor |
|  | CC_17L21 | Unsequenced |  | Acidic mammalian chitinase precursor |
|  | CC_24C24 | Unsequenced |  | Acidic mammalian chitinase precursor |
|  | CC_23M14 | gb|CA968087 | gi|50734238 | Acidic mammalian chitinase precursor |
| GE13c | CC_01C21 | Unsequenced |  | Carp Desaturase 2 (CDS2) |
|  | CC_03J07 | Unsequenced |  | Carp Desaturase 2 (CDS2) |
|  | CC_35M21 | Unsequenced |  | Carp Desaturase 2 (CDS2) |
| GE12 | CC_20B24 | Unsequenced |  | Troponin T, fast skeletal muscle isoforms |
|  | CC_26P05 | Unsequenced |  | Troponin T, fast skeletal muscle isoforms |
|  | CC_26A03 | gb|CA968139 | gi|50734290 | Troponin T, fast skeletal muscle isoforms |
| GE11 | CC_04L06 | Unsequenced |  | Apolipoprotein C-I precursor |
|  | CC_05I18 | Unsequenced |  | Apolipoprotein C-I precursor |
| GE10 | CC_15C13 | Unsequenced |  | Myoglobin |
|  | CC_04G20 | gb|CF662380 | gi|50741271 | Myoglobin |
|  | CC_14N14 | gb|CF662728 | gi|50741803 | Myoglobin |
|  | CC_28C23 | gb|CF662976 | gi|50742105 | Myoglobin |
|  | CC_30B20 | gb|CF660382 | gi|50742230 | Myoglobin |
| GE10b | CC_06G04 | Unsequenced |  | Warm-temperature-acclimation-related-65 kDa-protein |
|  | CC_06L20 | gb|CA965440 | gi|50738809 | Warm-temperature-acclimation-related-65 kDa-protein |
| GE10c | CC_05E20 | Unsequenced |  | Uncoupling protein 1 |
| GE9 | CC_03C21 | Unsequenced |  | C-type lectin |
| GE9c | CC_01I19 | Unsequenced |  | Elongation factor 1-alpha; EF-1-alpha |
|  | CC_02L14 | Unsequenced |  | Elongation factor 1-alpha; EF-1-alpha |
|  | CC_05O06 | Unsequenced |  | Elongation factor 1-alpha; EF-1-alpha |
| GE8 | CC_01M20 | Unsequenced |  | Alcohol dehydrogenase |
|  | CC_06L09 | Unsequenced |  | Alcohol dehydrogenase |
| GE5 | CC_13K22 | Unsequenced |  | RING finger protein 28 |
